# Assessment of improvements in exercise tolerance following pulmonary valve replacement using physical accelerometry

**DOI:** 10.1017/S1047951126113493

**Published:** 2026-06-08

**Authors:** Nicholas Joy, Jonathan H. Soslow, Kimberly Crum, Mary Killian, Sudeep Sunthankar

**Affiliations:** 1 Division of Pediatric Cardiology, Department of Pediatrics, Vanderbilt University Medical Center, USA; 2 Division of Pediatric Cardiology, Department of Pediatrics, University of Louisville School of Medicine, USA

**Keywords:** pulmonary valve replacement, exercise tolerance, accelerometry, tetralogy of Fallot

## Abstract

**Background:**

Pulmonary valve replacement may be required for patients with pulmonary valve disease. Exertional symptoms related to cardiovascular disease are an indication for pulmonary valve replacement; however, quantification of symptom improvement postoperatively is challenging, and previous studies have yielded mixed data. Accelerometry has the potential to remotely monitor changes in exercise tolerance following pulmonary valve replacement.

**Methods:**

Individuals (*n* = 18) age >13 years scheduled for catheter-based or surgical pulmonary valve replacement were prospectively enrolled. Participants were instructed to wear two Link GT9X accelerometers, placed on dominant wrist and one ankle, for seven days and nights before pulmonary valve replacement and again 3–6 months after the procedure.

**Results:**

The cohort’s median age was 26.5 [16.0, 35.0] years old. Compliance of both wrist and ankle accelerometers had a median of 96% or greater at both timepoints suggesting adequate participation. Accelerometry showed stability between participants. Quantitative analysis using pre-and post-pulmonary valve replacement accelerometer-derived measures yielded no significant differences. There was a significant decrease in the average score for items “Shortness of Breath with Strenuous Activity,” “Extent Symptoms Impact Daily Activities,” and “Challenges with Activities of Daily Living.” Furthermore, all 9 individuals with baseline exertional intolerance had resolution of symptom in postoperative assessment.

**Conclusion:**

Reliability of accelerometer-derived activity measurements supports usage of physical accelerometry as a remote outcome measure to reduce patient burden for onsite assessment. Mixed qualitative versus quantitative improvement in physical activity tolerance observed in this study aligns with previous studies on variable improvement in exercise tolerance following pulmonary valve replacement. This discrepancy suggests the need for a different quantitative mechanism to measure benefit.

## Introduction

Individuals with CHD particularly affecting the pulmonary valve and right ventricular outflow tract may require pulmonary valve replacement in adolescence or young adult years after primary corrective surgery.^1–9^ Indications for pulmonary valve replacement include imaging biomarkers, electrocardiogram changes, and clinical symptoms. Exercise intolerance, when not due to extracardiac disease, is considered an indication for pulmonary valve replacement; however, data regarding the exertional benefit following pulmonary valve replacement are sparse.^1–11^ Cardiopulmonary exercise testing found mixed improvement in percent predicted VO2 and VE/VCO2 following pulmonary valve replacement across several studies.^1,4–6,8,12–15^ The mixed data in these metrics demonstrate the need for a reliable measure to capture changes in exercise capabilities following pulmonary valve replacement.

Accelerometry provides a potential solution for a new outcome measure of exercise tolerance. Accelerometry has recently been utilised in several studies as a measure of daily physical activity levels in both individuals with heart failure and individuals with CHD.^16–22^ Potential advantages of accelerometry include utilisation in a remote, free-living environment and effort independence. This contrasts with standard measures such as cardiopulmonary exercise testing, which are usually effort dependent and must be performed in a clinical space.^1,8,12–14^

In this study, participants undergoing pulmonary valve replacement were assessed for improvements in a variety of clinical metrics including electrocardiogram, echocardiogram, cardiac MRI (cardiac MRI), cardiopulmonary exercise testing, and accelerometry. Our hypotheses were (1) accelerometry measured physical activity would increase following pulmonary valve replacement and (2) preoperative features (e.g., electrocardiogram and imaging metrics) would associate with the degree of improvement in activity level.

## Methods

### Participants and clinical measures

This study was approved by the Vanderbilt Institutional Review Board (IRB 211522). All procedures were performed in compliance with relevant laws and institutional guidelines. Privacy rights of human subjects were observed. Eligible participants were identified at time of scheduling pulmonary valve replacement and then were contacted separately to determine interest in participating in the study. Participant demographics and reason for procedure—including relevant surgical history—were documented at time of enrollment (Table A1). Individuals under the age of 13 were not eligible to participate. Participants over the age of 18 provided written informed consent, while participants under the age of 18 provided assent and parent or guardian informed consent. As available, participant preoperative and postoperative echocardiogram and electrocardiogram results were collected from the electronic medical record, and the variables selected for analysis are listed in the supplement (Table A2). Participants underwent either percutaneous or surgical valve replacement. Valve type and length of hospitalisation following replacement were collected via participant chart review.

### Accelerometry

Triaxial accelerometers (ActiGraph Link GT9X, Ametris LLC, Pensacola, FL) were utilised as a surrogate marker of daily preoperative physical activity. Prior to pulmonary valve replacement, participants were instructed to wear one accelerometer on their dominant wrist and one ankle accelerometer for 7 days, 24 hours a day, only removing to bathe. Post-pulmonary valve replacement accelerometer measurements were completed 3–6 months after pulmonary valve replacement, during which participants were again asked to wear an accelerometer on both wrist and ankle for 7 days, 24 hours a day. Compliance of wear was computed, taking the fraction of time accelerometer worn out of the 7-day period. Proportions of total activity spent in sedentary, light, and moderate-to-vigorous physical activity were computed as well as the number of activity counts, measured as vector magnitudes, per minute of wear. Accelerometer derived measures of entropy, jerk, and mean frequency were computed as described by Miller et al.^23^ Additional accelerometer parameter information is available in supplement (Supplementary Material 1).

### Quality of life survey

During both periods of accelerometry wear, participants also completed a self-report questionnaire assessing tolerance of daily physical activities. Questions concerning frequency of exercise-induced symptoms (including shortness of breath or fatigue) were scored using a response scale of never, rarely, sometimes, and frequently, while questions concerning challenges with daily activities (including self-care, housework, and general pain and discomfort) were scored using a scale of no problems, moderate problems, or extreme problems. Level of symptom severity was measured based on survey responses using the New York Heart Association classification system.^24^

### Statistical analysis

Wilcoxon signed rank tests were performed to assess any significant differences in continuous variables pre-and post-pulmonary valve replacement. A subgroup analysis was performed for participants with exercise intolerance symptoms as indication for pulmonary valve replacement. The subgroup was stratified by degree of severity based on baseline New York Heart Association classification. Spearman correlations were computed for associations between accelerometer-derived measures and clinical endpoints. Correlation strength was defined with *r* < 0.40 as weak, 0.40 < *r* < 0.70 as moderate, and *r* > 0.70 as strongly correlated as previously described.^25^ A threshold of *p* < 0.05 was used for significance. Paired t tests were run to assess significant changes in average score for participant-reported survey items.

## Results

A summary of participant demographic information is available in the supplement (Table A1). A total of 18 participants who underwent either transcatheter pulmonary valve replacement or surgical pulmonary valve replacement and completed both the pre-procedure and post-procedure portions of the study were included. At baseline assessment, the cohort’s median age was 26.5 [16.0, 35.0] years, and median body surface area was 1.73 [1.46, 1.93] m^2^ (Table 1). Twelve (67%) participants were male, and the most common primary diagnosis was tetralogy of Fallot (10/18, 59%). The majority of patients (89%) underwent transcatheter pulmonary valve replacement. Impaired exercise tolerance was reported in 50% (9/18) as a symptom at baseline, with none reporting the symptom’s persistence at clinic follow-up post-procedure.

For both ankle and wrist accelerometer wear across pulmonary valve replacement, median participant compliance was greater than 96% (Table 1). Paired median differences in key accelerometry and clinical assessment measures from pre-and post-pulmonary valve replacement were non-significant (Table 1). Sub-analyses isolating cohorts specifically reporting impaired exercise tolerance at baseline similarly showed no significant median differences (*n* = 9, Table A3) following pulmonary valve replacement.

Accelerometry measures of fraction of time spent in each activity level were highly correlated and all significant, as were measures of movement quality (entropy, jerk, and mean frequency). Most correlations between accelerometry measures and other clinical measures, including indexed right ventricular end-diastolic volume, QRS duration, left ventricular ejection fraction, and peak gradient pulmonary stenosis, were non-significant (Table A4). The correlation, however, between mean frequency and peak gradient pulmonary stenosis was moderately strong and positive (Table A4).

Most mean differences in patient-reported survey items pre-and post-pulmonary valve replacement were nonsignificant. Changes in score on the item “Engagement in Strenuous Activity” were highly variable. There was a significant decrease in the average score for the items “Shortness of Breath with Strenuous Activity,” “Extent Symptoms Impact Daily Activities,” and “Challenges with Activities of Daily Living” (Table 2).

## Discussion

In this prospectively enrolled single centre investigation of participants undergoing pulmonary valve replacement, evidence of postoperative improvement in exercise tolerance was mixed. We did not find a statistical difference in physical activity following pulmonary valve replacement using accelerometry metrics. Pulmonary valve replacement led to median reductions in peak gradient pulmonary stenosis following the procedure (Table 1); however, this did not confer an increase in quantitative activity measures. We observed 50% of the cohort recorded impaired exertional tolerance as a symptom prior to pulmonary valve replacement with all reporting resolution of this symptom following pulmonary valve replacement. This qualitative improvement is important, despite the lack of quantitative benefit.

Patient-described clinical symptoms are a key feature in the algorithm of determining when to perform pulmonary valve replacement.^26^ While exertional symptoms can be related to cardiovascular and non-cardiovascular aetiologies in individuals with CHD, the resolution of subjective symptoms within 6 months of pulmonary valve replacement leads the authors to hypothesise that pulmonary valve replacement was the primary contributor to patient benefit. Quality of life surveys in adults with tetralogy of Fallot, an anatomical population commonly requiring pulmonary valve replacement, demonstrate lower scores in domains related to physical activity compared to healthy controls; therefore, optimisation of subjective symptoms related to physical activity is a key outcome to consider.^27^ A meta-analysis including 48 studies and 3,118 patients with tetralogy of Fallot demonstrated a pooled reduction in New York Heart Association classification following pulmonary valve replacement, providing further evidence of the subjective benefit for postoperative patients.^28^

Despite the evidence of qualitative improvement, literature regarding quantitative improvement in exercise capacity following pulmonary valve replacement has been mixed. Previous studies analysing cardiopulmonary exercise testing data following pulmonary valve replacement showed mixed results with change in participant percent predicted VO2 max and VE/VCO2.^5,6,8,13,14,29^ In the two studies where participant percent predicted VO2 max significantly increased following pulmonary valve replacement, changes in respiratory exchange ratio were non-significant.^6,14^ The significant change in percent predicted VO2 max also did not correspond to the significant change in VE/VCO2 across studies. In this study, there were no significant improvements in the accelerometer-measured average fraction of activity time spent in more strenuous activity and total activity counts following pulmonary valve replacement. When considered with previous studies using cardiopulmonary exercise testing, these findings may further support the conclusion that exercise parameters do not significantly improve in individuals following pulmonary valve replacement. These mixed results between qualitative and quantitative measurements, in our own study, present a challenge in understanding if these data reflect a lack of true relationship or a need for more innovative exertional metrics to quantify improvement.

Accelerometer measurements have intrinsic limitations, particularly in data captured using the sensors placed on the ankle. The vector magnitudes per minute for participant ankles were less than participant wrists at both the pre-pulmonary valve replacement and post-pulmonary valve replacement timepoints (Table 1). Given that compliance of both wrist and ankle accelerometers had a median of 96% or greater at both timepoints, and that counts were indexed to the amount of wear time, it is less likely that compliance with the device was a major limitation (Table 1).

The representation of the study’s sample may have been further hindered by its range in baseline participant symptoms and age. Exertional symptoms were reported in 50% of the cohort at baseline survey; therefore, not all individuals were symptomatic prior to pulmonary valve replacement. Perhaps restricting inclusion criteria to those who were symptomatic and increasing sample size may have provided a cohort more likely to demonstrate a measured benefit. While a minimum age of 13 years was set, the 75^th^ percentile of participants analysed was 35 years old (Table A1). Although analyses were paired with participants in order to offset the random effects of this between-subjects variance, participant age may have been a mediating factor in the degree of improvement in exercise tolerance following pulmonary valve replacement. Further study would be required with a larger cohort to explore this potential mediation across specific age subgroups. Finally, limited availability of pre and post-pulmonary valve replacement participant cardiopulmonary exercise testing data hindered the ability to compare changes in accelerometry with other validated clinical measures. Only 6 participants had cardiopulmonary exercise testing data available at baseline, and none of the participants had post-pulmonary valve replacement cardiopulmonary exercise testing data to demonstrate changes. With mixed improvements across studies in cardiopulmonary exercise testing endpoints following pulmonary valve replacement, the availability of participant cardiopulmonary exercise testing data would have been a crucial reference point for the lack of significance in accelerometer-measured changes after pulmonary valve replacement.

Accelerometers have gained popularity in recent years as a novel outcome measure in CHD studies.^19,20, 30,31^ Given the focus on exertional symptoms as an indication for pulmonary valve replacement,^26^ these patients, and particularly those with tetralogy of Fallot, have been perceived as a population in which accelerometers may have utility. While our sample size was modest, it was adequate to detect a clinically significant improvement in accelerometer measures following pulmonary valve replacement. Lack of this signal suggests a different modality may better quantify activity changes and correlate with our detected subjective improvement.

## Conclusion

Subjective impaired exertional intolerance resolved following pulmonary valve replacement; however, quantitative measure of physical activity by accelerometry did not show significant changes. While physical accelerometry has the potential to serve as a free-living physical capacity measure, our data suggest investigation into alternative quantitative measures may be required given the discrepancy with qualitative data.

## Supplementary Material

1

The supplementary material for this article can be found at https://doi.org/10.1017/S1047951126113493.

## Figures and Tables

**Table 1.** Median differences in key outcome measures (*N* =18)

Measure	Pre-PVR median [IQR]	Post-PVR median [IQR]	*p*-Value
VMs Per Min (Wrist)	1768.41 [1553.99, 2021.73]	1669.76 [1472.53, 2077.79]	0.325
Wear compliance (Wrist)	0.99 [0.97. 1.00]	0.97 [0.95, 1.00]	0.224
VMs Per Min (Ankle)	723.05 [502.40, 816.68]	767.09 [550.38, 1006.17]	0.359
Wear compliance (Ankle)	0.98 [0.96, 1.00]	0.96 [0.94, 0.98]	0.021
FOT sedentary	0.80 [0.76, 0.84]	0.81 [0.77, 0.85]	0.284
FOT low intensity	0.18 [0.15, 0.20]	0.17 [0.14, 0.21]	0.393
FOT MVPA	0.02 [0.02, 0.03]	0.02 [0.01, 0.03]	0.523
Entropy	0.66 [0.54, 0.79]	0.74 [0.57, 0.87]	0.298
Average jerk	0.82 [0.67, 0.97]	0.80 [0.62, 0.99]	0.065
Mean frequency	3.28 [3.17, 3.33]	3.29 [3.19, 3.41]	0.934
QRS duration (ms)	110.50 [100.00, 132.00]	108.00 [98.00, 145.00]	0.925
LVEF (%)	57.00 [55.00, 63.00]	58.65 [56.10, 61.90]	0.760
Peak gradient PS (mmHg)	37.50 [24.00, 62.00]	21.00 [16.00, 25.00]	0.009

Accelerometer-derived measures and clinical outcomes before and after pulmonary valve replacement.

**Table 2.** Mean differences in patient-reported questionnaire items (*N* =18)

Question	Average score change	*p*-Value
Shortness of breath on stairs	−0.500	0.132
Engagement in strenuous activity	0	0.999
Shortness of breath in strenuous activity	−0.444	0.042
Distance (Blocks) able to walk in normal conditions	0.500	0.095
Shortness of breath in sedentary activity	−0.167	0.331
Extent symptoms impact daily activities	−0.833	0.014
Challenges with self-care	−0.111	0.331
Challenges with activities of daily living	−0.333	0.029
Regular pain or discomfort	−0.111	0.495
Anxiety or depression	−0.056	0.668

All survey items except for distance walked are reverse scored, with lower scores reflecting more affirmative.

**Table A1.** Demographics of cohort included for analysis (*N* = 18)

Demographic	Median [IQR] or count (%)
Age at PVR referral (yrs)	26.50 [16.00, 35.00]
Male	12 (66.7%)
Height (cm)	168.80 [157.00, 173.00]
Weight (kg)	70.45 [54.40, 76.20]
BSA	1.73 [1.46, 1.93]
Primary diagnosis tetralogy of Fallot	10 (58.8%)
Exertional symptoms as indication for PVR	9 (50.0%)

Anthropometrics summarised at baseline (pre-PVR).

PVR = pulmonary valve replacement.

**Table A2.** Summary of variables analysed from participant clinical measures

Clinical assessment	Variable analysed (units)
Echocardiogram	LVEF (%)
	Peak gradient pulmonary stenosis (mmHg)
EKG	QRS duration (ms)
	Presence of arrhythmia

Parameters were captured both retrospectively (pre-PVR) and prospectively (post-PVR).

Preoperative cardiac MRI data were also collected for participants with available scan, but was excluded from analysis due to limited data capture (*n* = 8 participants).

LVEF = left ventricular ejection fraction; MRI = magnetic resonance imaging.

**Table A3.** Median differences in participants indicated for PVR by exercise intolerance (*N* = 9)

Measure	NYHA < 3 (*N* = 5)			NYHA ≥ 3 (*N* = 4)		
	Pre-PVR Median [IQR]	Post-PVR Median [IQR]	*P*-Value	Pre-PVR Median [IQR]	Post-PVR Median [IQR]	*P*-Value
VMs per Min (wrist)	1872.79 [1628.90, 2021.73]	1705.54 [1472.53, 1877.33]	0.125	1769.79 [1574.14, 2078.46]	1790.18 [1444.12, 2295.99]	0.875
VMs per Min (ankle)	837.42 [502.40, 1005.89]	880.86 [340.47, 907.18]	0.750	486.13 [375.33, 595.09]	651.16 [518.33, 767.09]	0.500
FOT sedentary	0.79 [0.75, 0.83]	0.81 [0.77, 0.85]	0.063	0.80 [0.76, 0.82]	0.79 [0.73, 0.83]	0.875
FOT low intensity	0.18 [0.16, 0.22]	0.17 [0.14, 0.20]	0.063	0.18 [0.16, 0.20]	0.18 [0.15, 0.23]	0.875
FOT MVPA	0.02 [0.01, 0.03]	0.02 [0.01, 0.02]	0.188	0.03 [0.02, 0.04]	0.03 [0.01, 0.04]	0.375
Entropy	0.56 [0.54, 0.75]	0.64 [0.46, 0.78]	0.875	0.71 [0.54, 0.87]	0.87 [0.70, 1.00]	0.125
Average jerk	0.82 [0.73, 0.94]	0.69 [0.57, 0.87]	0.125	0.74 [0.65, 0.92]	0.74 [0.62, 0.91]	0.875
Mean frequency	3.25 [3.11, 3.30]	3.42 [3.18, 3.56]	0.250	3.26 [3.21, 3.34]	3.19 [3.14, 3.27]	0.625
QRS duration (ms)	103.00 [99.00, 108.00]	124.00 [101.50, 156.50]	0.250	128.00 [121.50, 154.50]	127.00 [109.00, 158.00]	0.875
LVEF (%)	57.50 [53.50, 67.30]	60.00 [55.00, 71.10]	0.999	55.00 [54.00, 55.00]	55.00 [55.00, 69.20]	0.999
Peak gradient PS (mmHg)	39.00 [36.90, 62.50]	17.00 [16.00, 32.50]	0.181	24.00 [12.00, 36.00]	12.10 [11.20, 17.60]	0.500

Median differences in accelerometer-derived measures and clinical outcomes from before and after pulmonary valve replacement for those who exercise intolerance were reported as an indication for PVR. The analysis was stratified by symptom severity, with NYHA class < 3 established as less severe exercise intolerance and ≥ 3 as more severe.

LVEF = left ventricular ejection fraction; MVPA = moderate-to-vigorous physical activity; NYHA = New York Heart Association; PVR = pulmonary valve replacement.

**Table A4.** Correlations of key outcome measures (*N* = 18)

	QRS duration	LVEF	Peak gradient PS
VMs per Min (wrist)	*r* = −0.15^ns^	*r* = −0.26^ns^	*r* = −0.11^ns^
VMs per Min (ankle)	*r* = −0.22^ns^	*r* = 0.07^ns^	*r* = −0.16^ns^
FOT sedentary	*r* = 0.18^ns^	*r* = 0.35^ns^	*r* = 0.12^ns^
FOT light activity	*r* = −0.22^ns^	*r* = −0.35^ns^	*r* = −0.12^ns^
FOT MVPA	*r* = −0.12^ns^	*r* = −0.22^ns^	*r* = −0.08^ns^
Entropy	*r* = −0.18^ns^	*r* = −0.27^ns^	*r* = −0.22^ns^
Average jerk	*r* = −0.12^ns^	*r* = 0.12^ns^	*r* = 0.04^ns^
Mean frequency	*r* = 0.12^ns^	*r* = 0.15^ns^	*r* = 0.43*

Key for significance:

ns : *P* > 0.05

* : *P* < 0.05

** : *P* < 0.01

*** : *P* < 0.001.

MVPA = moderate-to-vigorous physical activity.
