# Supplementary material for: Assessment of improvements in exercise tolerance following pulmonary valve replacement using physical accelerometry
Source: Cardiol Young. Author manuscript; Available in PMC 2026 Aug 4. (PMC13311448; doi:10.1017/S1047951126113493)
Supplement: 1 [file NIHMS2175068-supplement-1.docx]

Assessment of improvements in exercise tolerance following pulmonary valve replacement using physical accelerometry

Nicholas Joy, BS^1^, Jonathan Soslow, MD, MSCI^1^, Kimberly Crum, RN^1^, Mary Killian, MD^1^, Sudeep Sunthankar, MD, MSCI^1^

1. Division of Pediatric Cardiology, Department of Pediatrics, Vanderbilt University Medical Center, Nashville, TN

Corresponding Author: Nicholas Joy; nicholas.joy@vumc.org

Accelerometer data was collected at a frequency of 30 Hz (30 data points per second per axis). For data processing, the raw accelerometry data was first classified into wear and non-wear time according to Choi’s algorithm^[1]^ integrated into 15 second epochs, and then converted into rooted-squared-sum vector magnitude (VM) counts using ActiLife software (Ametris, Pensacola, FL). Days lacking at least 600 minutes of accelerometer-defined wear time between 07:00 and 22:00 (“daytime”), as well as 300 minutes between 22:00 and 07:00 (‘nighttime”), were not considered valid days and were excluded from analysis^[1,2]^ Accelerometer wear periods without 1 valid day of wear were excluded from analysis. Proportions of total activity spent in sedentary, light, and moderate-to-vigorous physical activity (MVPA) were also calculated for the wrist using previously identified VM cutpoints^[3]^. To compute movement quality variables of sample entropy, jerk, and mean frequency, raw 30 Hz data and down-sampled 1 Hz data were first extracted using ActiLife software and filtered using Ametris’ proprietary algorithm^[4]^. The variables of interest were then computed using custom R (v. 4.4.2) code, with entropy being calculated from the 1 Hz data while jerk and mean frequency were calculated from the 30 Hz data. The R code is available on <https://github.com/keithlohse/HarmonizedAccelData> and archived on Zenodo^[5]^.

Supplementary Material 1. Expanded description of accelerometer processing and measure derivation. Cited references include:

1. Choi L, Liu Z, Matthews CE, Buchowski MS. Validation of Accelerometer Wear and Nonwear Time Classification Algorithm. Medicine & Science in Sports & Exercise. 2011 Feb;43(2):357–64. doi: 10.1249/MSS.0b013e3181ed61a3

2. Joy N, Soslow J, Burnette WB, et al (2026) Six-Minute Activity-95th Centile, a Novel Wearable-Derived Clinical Outcome Assessment for Duchenne Muscular Dystrophy. Pediatric Neurology 175:187–195. doi: 10.1016/j.pediatrneurol.2025.11.017

3. Arteaga D, Donnelly T, Crum K, Markham L, Killian M, Burnette WB, et al. Assessing Physical Activity Using Accelerometers in Youth with Duchenne Muscular Dystrophy. J Neuromuscul Dis. 2020;7(3):331–42. doi: 10.3233/JND-200478

4. Miller AE, Lohse KR, Bland MD, Konrad JD, Hoyt CR, Lenze EJ, et al. A Large Harmonized Upper and Lower Limb Accelerometry Dataset: A Resource for Rehabilitation Scientists [Preprint]. medRxiv; 2024;p. 2024.08.15.24312066. Available from: doi: 10.1101/2024.08.15.24312066

5. Lohse KR. keithlohse/HarmonizedAccelData: Harmonized Upper and Lower Limb Accelerometry Data. 2024.
